# Case Report: Abnormal ECG in a Patient With Acute Pancreatitis

**DOI:** 10.3389/fcvm.2021.741253

**Published:** 2021-12-23

**Authors:** Yunxiang Long, Manyun Tang, Jie Wang, Hui Liu, Zhijie Jian, Guoliang Li, Chang Liu

**Affiliations:** ^1^Department of Hepatobiliary Surgery, The First Affiliated Hospital of Xi'an Jiaotong University, Xi'an, China; ^2^Department of Cardiovascular Medicine, The First Affiliated Hospital of Xi'an Jiaotong University, Xi'an, China; ^3^Department of Hematology, The First Affiliated Hospital of Xi'an Jiaotong University, Xi'an, China; ^4^The Biobank of the First Affiliated Hospital of Xi'an Jiaotong University, Xi'an, China; ^5^Department of Radiology, The First Affiliated Hospital of Xi'an Jiaotong University, Xi'an, China

**Keywords:** acute abdomen, diagnosis, coronary computed tomography angiography, ST-segment elevation myocardial infarction, case report

## Abstract

**Background:** Both acute pancreatitis and acute myocardial infarction (AMI) are rapidly progressive and frequently fatal diseases that can be interrelated and lead to a vicious cycle for further problems. The concomitant occurrence of AMI and acute pancreatitis is rare but critical, and efficient diagnosis and treatment of such patients are challenging.

**Case Summary:** We reported an uncommon case of abnormal ECG findings in a 63-year-old woman with acute pancreatitis. The patient exhibited increased biomarkers of myocardial injury, such as creatine kinase-MB (CK-MB) and troponin T, as well as ST segment elevation in inferior leads II, III, and aVF. Both of these have been previously observed in patients with acute abdomen in the absence of ST-segment elevation myocardial infarction (STEMI), including pancreatitis. In addition, lacking complaints of chest pain or tightness was also supportive of this idea. Echocardiography indicated abnormalities in the functioning of the left inferior posterior wall segments and decreased overall systolic function of the left ventricle with a 51% ejection fraction. Eventually, AMI was diagnosed after coronary computed tomography angiography (CCTA) showing critical stenosis of the right coronary artery and left anterior descending artery segments. The patient was urgently transferred to intensive care unit and was treated with anticoagulation, antiplatelet aggregation, lipid-lowering and other palliative drugs.

**Conclusion:** Concomitant acute pancreatitis and AMI are often considered to be critical conditions with a poor prognosis. Therefore, it is important to rapidly identify this condition and consider transferring patients for multidisciplinary supportive care.

## Introduction

Acute abdomen, including acute pancreatitis, can often be accompanied by various ECG changes, such as arrhythmias, dynamic T waves, pathologic Q waves and ST segment elevation, although the underlying mechanisms remain unknown (1). ST segment elevation is often transient and returns to normal after the acute condition is stabilized, which does not indicate AMI (2, 3). Although acute pancreatitis and concomitant myocardial infarction are uncommon in patients, we should be vigilant when patients with acute pancreatitis experience ST segment elevation in ECG because it often results in a poor prognosis and requires urgent treatment.

## Case Report

A 63-year-old woman was admitted to emergency department of the local hospital due to persistent abdominal pain accompanied by nausea and vomiting for 2 days. She denied chest pain or tightness. She had a medical history of hypertension and diabetes over the past 20 years. Four years prior, she was diagnosed with gallbladder stones, cholecystitis and pancreatitis, and surgery was recommended; however, the patient refused. She denied smoking and drinking.

Emergency abdominal CT in the local hospital indicated the possibility of pancreatitis. After the patient was transferred to our emergency department, her vital signs stabilized. On physical examination, there was diffuse tenderness in her abdomen, especially in the left upper abdominal quadrant, without obvious rebound tenderness or abdominal muscle tension.

**Figure 1:**
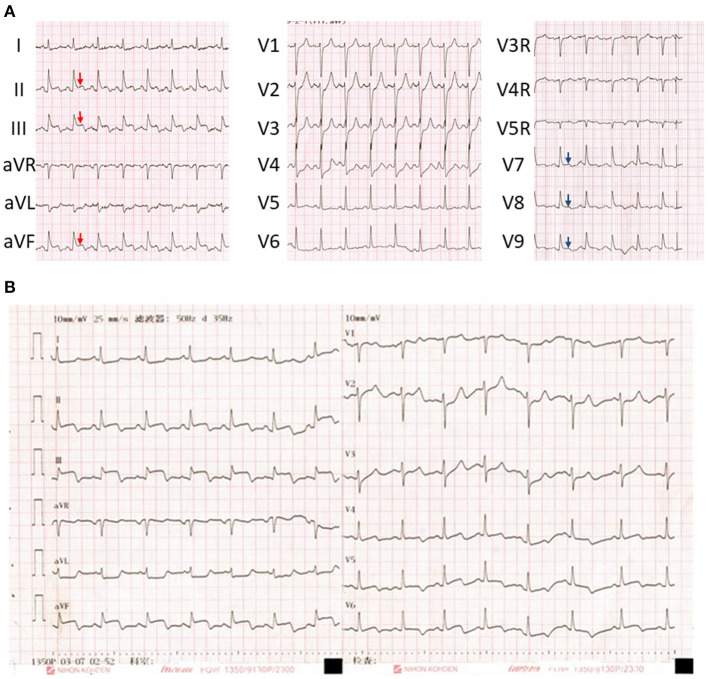
**(A)** Initial electrocardiogram on admission showed ST segment elevation in the inferior leads II, III, and aVF (red arrows) and posterior wall (blue arrows). **(B)** a dynamic ECG pattern was also observed on the second after admission.

The admission ECG (Figure 1A) showed ST segment elevation in inferior leads II, III, and aVF, reciprocal depression in leads V1-4 and elevation in posterior leads. In addition, a dynamic ECG pattern was also observed on the second after admission (Figure 1B). These ECG changes were consistent with the electrocardiographic findings of STEMI, and this interpretation was further supported by the subsequently elevated biomarkers of myocardial injury. Furthermore, echocardiography indicated abnormal movement of left inferior posterior wall segments and decreased overall systolic function of left ventricle with a 51% ejection fraction.

**Figure 2:**
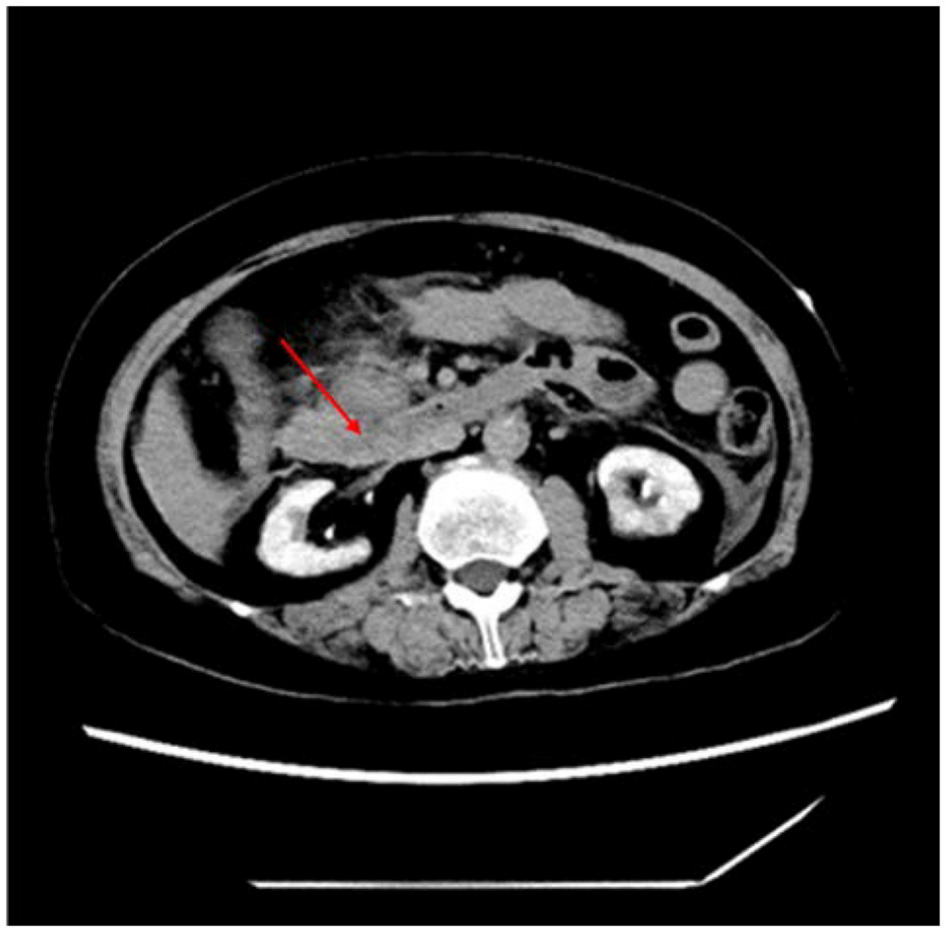
Abdominal CT on admission showed acute pancreatitis with peripancreatic exudation (red arrows).

**Figure 3:**
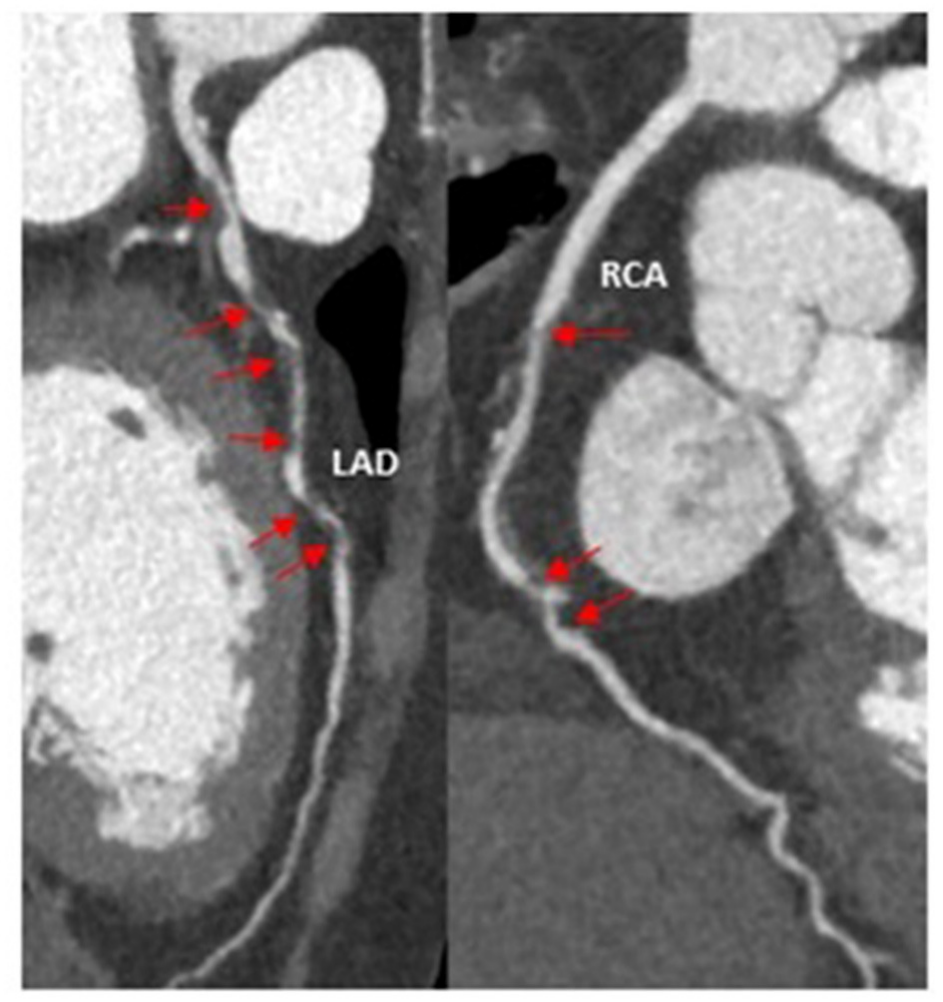
Coronary computed tomography angiography indicated critical stenosis (red arrows) of right coronary artery and left anterior descending coronary artery.

Repeated abdominal CT (Figure 2) combined with the marked elevation of serum amylase (886 U/L, reference range, 35–135 U/L) and lipase (3,451 U/L, reference range, 23–300 U/L) in our hospital indicated a definitive diagnosis of acute pancreatitis and multiple atherosclerosis. It is important to note that the biomarkers of myocardial injury were five times higher than the normal upper limit: CK-MB was 210.8 U/L (reference range, 0–24 U/L) and troponin T was 6.910 ng/mL (reference range, 0–0.014 ng/mL). CCTA (Figure 3) was performed after informed consent was obtained, which indicated severe stenosis with non-calcified plaques of multiple coronary arteries, including the third segment of right coronary artery and the sixth and seventh segments of left anterior descending artery. Therefore, acute inferior myocardial infarction was definitively diagnosed.

Given the poor outcomes of concomitant AMI and acute abdomen, the patient was urgently transferred to intensive care unit and was treated with anticoagulation, antiplatelet aggregation, lipid-lowering and other palliative drugs. Three days after admission, however, the patient unfortunately developed progressive multiple-organ failure, and resuscitation was not successful.

## Discussion

Based on the imaging examination and biomarkers, acute pancreatitis was reliably diagnosed in this case. The major challenge, however, was what the abnormal ECG findings implied. The observed ECG pattern (Figure 1) and simultaneous biomarkers of myocardial injury, both of which are always suggestive of STEMI, were also previously observed in patients with acute abdomen alone, including pancreatitis (2, 4). Therefore, the diagnosis of STEMI remained elusive on admission. However, the uncommon condition in which acute pancreatitis and AMI concomitantly occur has also been reported (5). Echocardiography and CCTA are crucial parts of the evidence chain and the key to correct diagnosis. Therefore, given the possibility of AMI induced by acute pancreatitis, the typical ECG pattern of STEMI, abnormal left ventricular wall movement on echocardiography, elevated biomarkers of myocardial injury, and CCTA showing critical stenosis of multiple coronary arteries, the patient was finally diagnosed with concomitant STEMI and acute pancreatitis. In addition, multiple atherosclerosis observed on abdominal CT examination also supported the specific outcome of AMI. Based on systemic atherosclerosis, trypsin damage and high blood flow, stiff coronary arteries are more likely to become further narrowed or blocked.

ECG has moderate sensitivity and specificity for detecting AMI (42% and 65%, respectively) (6). The working diagnosis of STEMI is usually based on symptoms consistent with myocardial ischemia, including persistent chest pain and new ST segment elevation (7). However, a few cases have reported that ST segment elevation does not contribute to AMI or Takotsubo cardiomyopathy, such as ventricular aneurysm (8), acute pericarditis (9), variant angina (10), septic shock (11, 12) and acute myocarditis (13). The possible causes of gastrointestinal symptoms include perforated gastric ulcer, intestinal obstruction, acute appendicitis, acute pancreatitis, and acute cholecystitis. Furthermore, these can also coexist with AMI (14). To mitigate the misdiagnosis of STEMI, echocardiography and CCTA should be undertaken to identify the causes of myocardial ischemia, especially in conditions of concomitant acute abdomen.

In this work, the patient was eventually diagnosed with concomitant AMI and acute pancreatitis. However, a larger proportion of reported cases of acute pancreatitis with ST segment elevation were not diagnosed with STEMI. Amgad et al. presented a patient with acute pancreatitis whose ECG occurred ST segment elevation in the inferior wall leads, but coronary artery lesions were not remarkable (15). Similarly, Effoe et al. reported an acute pancreatitis case with epigastric pain and left chest pain radiating to the left arm. Series electrocardiograms showed ST segment elevation and dynamic T wave changes in the right precordial electrocardiogram leads, which was consistent with Wellens syndrome. However, the patient's coronary arteriography was normal. After aggressive treatment for acute pancreatitis, the patient's chest pain and abnormal ECG changes returned to normal (16). Yu also summarized 36 cases of acute pancreatitis with simulated AMI in the previous literature, among which the inferior wall STEMI pattern was the most common (1). In summary, acute pancreatitis was associated with the STEMI electrical pattern with normal coronary arteries. Therefore, ST segment elevation, in this case, was initially suspected as the secondary ECG pattern induced by acute pancreatitis rather than STEMI.

ST segment elevation and elevated biomarkers of AMI are often observed in patients with acute pancreatitis in the absence of AMI (1, 5). First, trypsin can damage blood vessels, promote platelet aggregation and thrombosis, and stimulate the production of vasoactive substances such as bradykinin. Trypsin can also directly damage myocardium and inhibit myocardial contraction (5, 17). Second, inflammatory mediators play an essential role in myocardial infarction. Inflammatory cells in atherosclerotic plaques can be activated by circulating inflammatory cytokines, further resulting in plaque instability. In addition, inflammation and stress can increase the metabolic needs of myocardial cells, which can exceed the capacity of blood to supply oxygen to cells, especially in coronary stenosis (18, 19). This reminds us that “pancreatitis-related ECG and biomarker abnormalities” is still a diagnosis of exclusion only after excluding coronary artery disease, even in cases without the syndrome of chest pain or tightness (20).

Determining the preferred treatment strategy is another challenge. Acute pancreatitis, a serious illness due to various complications, is an absolute contraindication to emergency percutaneous coronary intervention (PCI). Moreover, it seems that AMI occurred 2 days before admission, clearly out of the window of primary PCI. Furthermore, AMI is in turn an absolute contraindication to exploratory laparotomy due to high risk of major adverse cardiovascular events (21). Therefore, conservative treatment with antithrombotic and lipid-lowering drugs was chosen.

In summary, we presented an uncommon case with concomitant acute pancreatitis and AMI. Although ST segment elevation and elevated biomarkers of myocardial injury are often observed in the acute abdomen, “pancreatitis-related ECG and biomarker abnormalities” is still a diagnosis of exclusion only after excluding coronary artery disease. It is of vital importance for cardiologists as well as emergency physicians, ambulance staff, and other caregivers involved in the triage of these patients to be well-trained to rapidly identify this situation and consider transferring patients for urgent and appropriate therapy. However, we regretted that autopsy was not performed due to the refuse of patient's family, despite that it is vital to confirm AMI by autopsy in this case.

## Data Availability Statement

The original contributions presented in the study are included in the article/Supplementary Material, further inquiries can be directed to the corresponding author/s.

## Ethics Statement

Written informed consent was obtained from the individual(s), and minor(s)' legal guardian/next of kin, for the publication of any potentially identifiable images or data included in this article.

## Author Contributions

YL, MT, and JW contributed to conception and design of the study. HL organized the database. YL and ZJ wrote the first draft of the manuscript. YL, GL, and CL wrote sections of the manuscript. All authors contributed to the article and approved the submitted version.

## Funding

This work was supported by the Clinical Research Award of the First Affiliated Hospital of Xi'an Jiaotong University, China (XJTU1AF-CRF-2018-015).

## Conflict of Interest

The authors declare that the research was conducted in the absence of any commercial or financial relationships that could be construed as a potential conflict of interest.

## Publisher's Note

All claims expressed in this article are solely those of the authors and do not necessarily represent those of their affiliated organizations, or those of the publisher, the editors and the reviewers. Any product that may be evaluated in this article, or claim that may be made by its manufacturer, is not guaranteed or endorsed by the publisher.
