# Supplementary figures and images for: Case Report: Abnormal ECG in a Patient With Acute Pancreatitis
Source: Front Cardiovasc Med. 2021 Dec 23;8:741253. doi: 10.3389/fcvm.2021.741253 (PMC8733163; doi:10.3389/fcvm.2021.741253)

## Slide 1
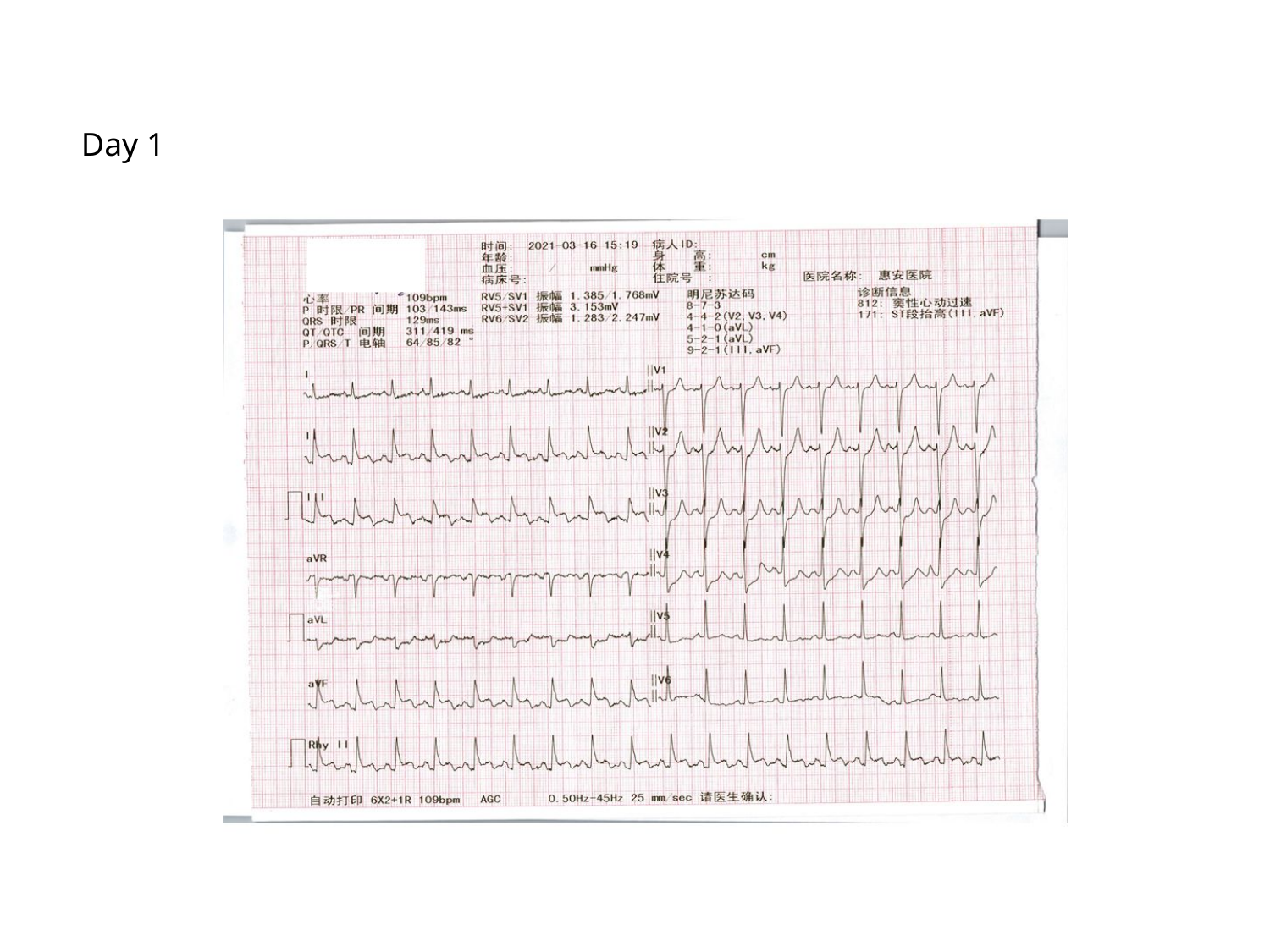

Day 1

## Slide 2
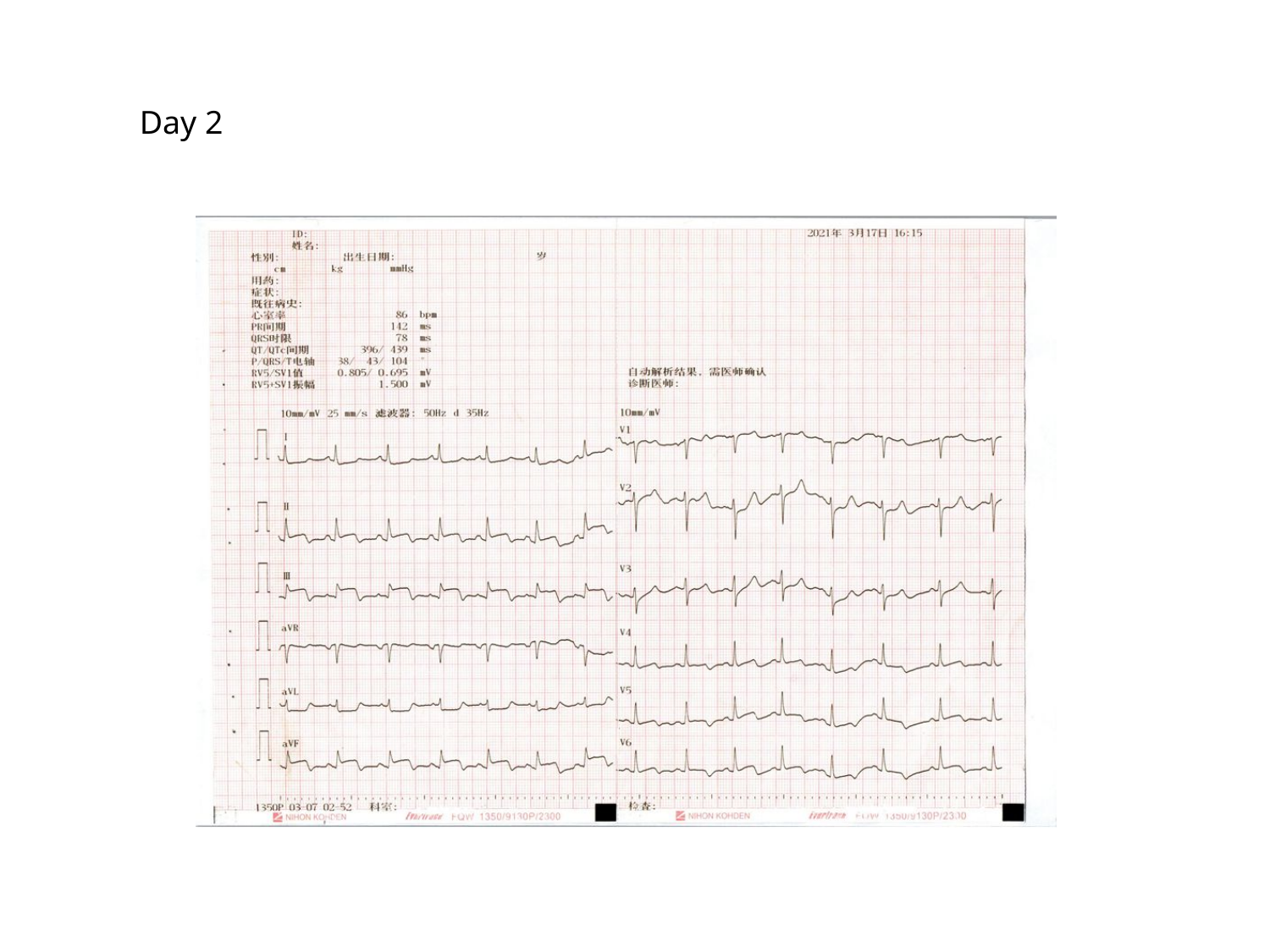

Day 2

## Slide 3
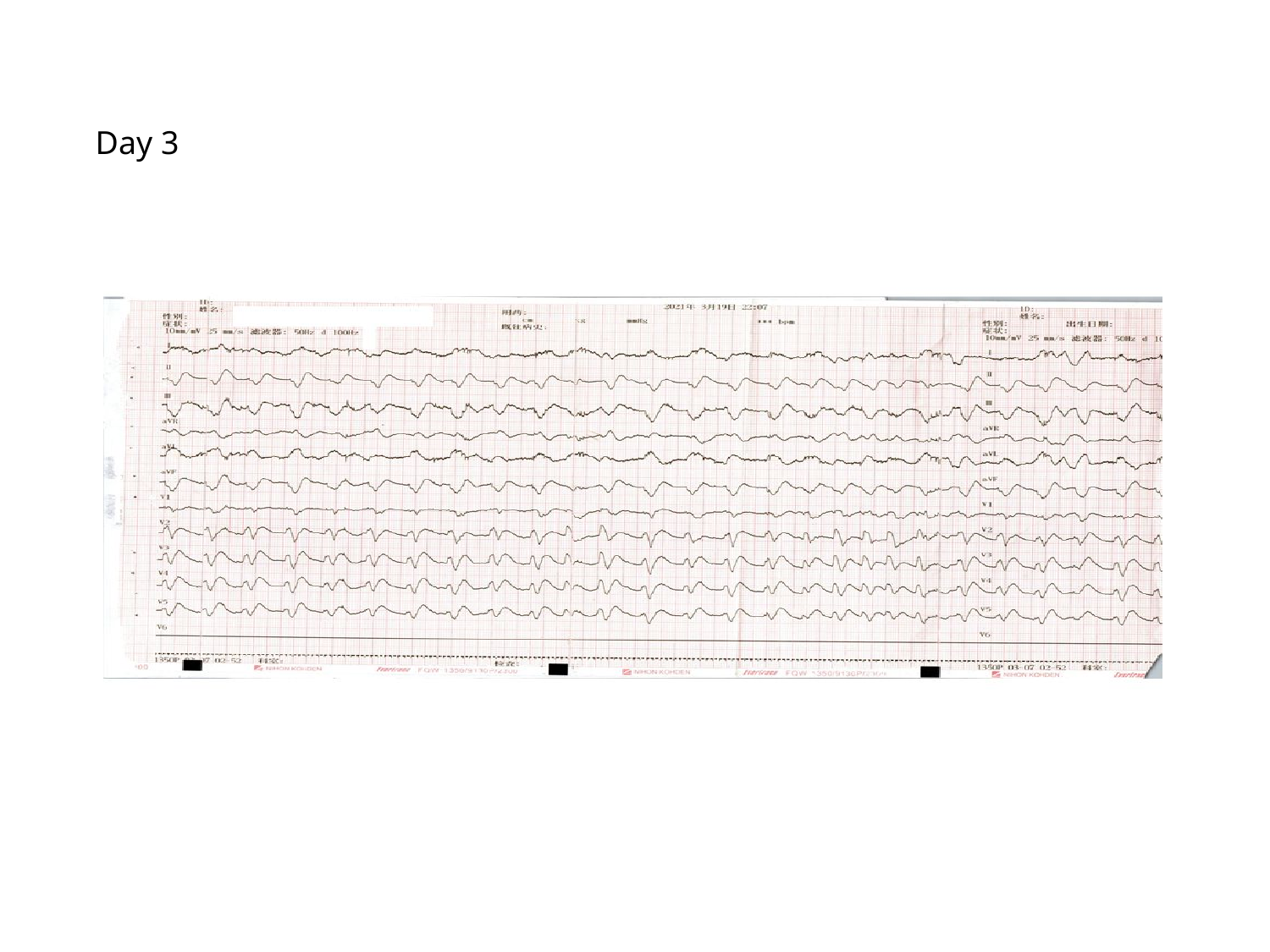

Day 3

Supplement: Supplementary file 1 [file Presentation_1.PPTX]
